# The impact of COVID-19 on the care of people living with noncommunicable diseases in low- and middle-income countries: an online survey of physicians and pharmacists in nine countries

**DOI:** 10.1017/S146342362100030X

**Published:** 2021-06-14

**Authors:** Chris Bullen, Jessica McCormack, Amanda Calder, Varsha Parag, Kannan Subramaniam, Anurita Majumdar, Pai-Hui Huang, Ratna Devi, Luna El Bizri, Felicity Goodyear-Smith

**Affiliations:** 1 National Institute for Health Innovation, University of Auckland, Auckland, New Zealand; 2 Medical Affairs and Clinical Research, Viatris Ltd, Auckland, New Zealand; 3 Medical Affairs and Clinical Research, Pfizer PFE Private Limited (A Viatris Company), Singapore; 4 Medical Affairs and Clinical Research, Viatris Taiwan, Taipei, Taiwan; 5 International Alliance of Patient’s Organizations, IAPO, London, UK; 6 Higher Institute of Public Health, St Joseph’s University, Beirut, Lebanon; 7 Professor of General Practice and Primary Health Care, University of Auckland, Auckland, New Zealand

**Keywords:** COVID-19, noncommunicable diseases, online survey, pharmacists, general practitioners, primary care

## Abstract

**Background:**

The global COVID-19 pandemic has disrupted healthcare worldwide. In low- and middle-income countries (LMICs), where people may have limited access to affordable quality care, the COVID-19 pandemic has the potential to have a particularly adverse impact on the health and healthcare of individuals with noncommunicable diseases (NCDs). A World Health Organization survey found that disruption of delivery of healthcare for NCDs was more significant in LMICs than in high-income countries. However, the study did not elicit insights into the day-to-day impacts of COVID-19 on healthcare by front-line healthcare workers (FLHCWs).

**Aim:**

To gain insights directly from FLHCWs working in countries with a high NCD burden, and thereby identify opportunities to improve the provision of healthcare during the current pandemic and in future healthcare emergencies.

**Methods:**

We recruited selected frontline healthcare workers (general practitioners, pharmacists, and other medical specialists) from nine countries to complete an online survey (n = 1347). Survey questions focused on the impact of COVID-19 pandemic on clinical practice and NCDs; barriers to clinical care during the pandemic; and innovative responses to the many challenges presented by the pandemic.

**Findings:**

The majority of FLHCWs responding to our survey reported that their care of patients had been impacted both adversely and positively by the public health measures imposed. Most FLHCs (95%) reported a deterioration in the mental health of their patients.

**Conclusions:**

Continuity of care for NCDs as part of pandemic preparedness is needed so that chronic conditions are not exacerbated by public health measures and the direct impacts of the pandemic.

## Introduction

An estimated 41 million people worldwide die from noncommunicable diseases (NCDs) each year (World Health Organization, 2018). The pandemic has disrupted millions of lives, with the SARS-CoV-2 virus infecting (at December 15, 2020) more than 71 million people worldwide and causing more than 1.6 million deaths (World Health Organization, 2021a). If infected, people living with NCDs are at higher risk than those without NCDs of developing severe COVID-19-related illness and death (World Health Organization, 2020a). NCD care in healthcare systems, already limited in capacity, has been under further stress, as resources and personnel have been diverted toward control and management of the outbreak.

The burden of NCDs falls disproportionately on people living in LMICs, with more than 85% of premature deaths due to NCDs occurring in low- and middle-income countries (LMICs) (World Health Organization). Effective prevention and management of NCDs may not be possible in LMICs because of the continuing call on resources to prevent and manage infectious diseases and acute conditions (Haque *et al.*, 2020). Additionally, broader environmental, political, and social conditions may not be conducive to health promotion activities (Allotey *et al.*, 2014).

Primary care plays a vital role in the prevention and management of NCDs (World Health Organization, 2021a), for example, in identifying risk factors and providing brief interventions (Beaglehole *et al.*, 2008). Lack of access to primary healthcare services can contribute to and exacerbate the acquisition of NCDs (Haque *et al.*, 2020). In LMICs, universal access to affordable quality care of NCDs is achievable only through effective primary care services, such as those provided by frontline health care workers – community health workers, nurses, family physicians/general practitioners, and community pharmacists (Joint Learning Innitiative, 2004).

### NCDs during pandemics and natural disasters

During emergencies, routine care provision and on-going management of NCDs may be further disrupted (Ochi *et al.*, 2014). Continuity of healthcare may also be impacted by failures in the supply chain of equipment and medications, closure or evacuation of healthcare services (Arrieta *et al.*, 2008), and reduced attendance and preparedness by frontline healthcare workers (Jaakkimainen *et al.*, 2014, Balicer *et al.*, 2006). Infection control measures, such as quarantine and rationing of emergency supplies, may contribute to the disruption of short-term healthcare (Elston *et al.*, 2017; Jaakimainen *et al.*, 2014). Likewise, emergencies and natural disasters can lead to lower rates of clinic attendance by patients (Kelly *et al.*, 2015) and lower levels of medication management and adherence (Ochi *et al.*, 2014). Since the start of the COVID-19 pandemic, similar experiences have been reported from a range of healthcare systems (Chang *et al.*, 2020, Palmer *et al.*, 2020, Prescott *et al.*, 2020). The impact of emergencies and pandemics on NCDs is more severe in LMICs (Slama *et al.*, 2017). On-going management of the crisis exacerbates preexisting healthcare vulnerabilities in capacity and capability (Kraef *et al.*, 2020, Sharma *et al.*, 2020, Siedner *et al.*, 2020). Consequently, people with NCDs, already more susceptible to becoming seriously ill if they become infected with the SARs-CoV-2 virus, may be less able to access adequate treatment to manage their NCD.

In a recent survey of Ministries of Health in 163 member states, the World Health Organization (WHO) found that the COVID-19 pandemic had severely interrupted prevention and treatment services for NCDs: 122 countries reported service disruptions due to the pandemic (World Health Organization, 2020b). More than half (53%) of the countries surveyed reported having partly or completely disrupted services for hypertension treatment, 49% for diabetes and diabetes-related complications, and 31% for cardiovascular emergencies. In 94% of countries, the Ministry of Health staff working in NCDs were partly or fully reassigned to support the COVID-19 response. Population health screening programs have also been interrupted, with most significant disruption in countries experiencing high levels of community transmission (World Health Organization, 2020a).

Thus far, research has failed to capture insights into the day-to-day impacts of COVID-19 on FLHCWs (including medical doctors, registered nurses, community pharmacists, and state registered allied staff) providing healthcare for NCDs. In this study, we aimed to gain insights directly from FLHCWs, specifically doctors and pharmacists, working in countries with a high NCD burden, and thereby, identify opportunities to improve the provision of healthcare during the current pandemic and in future healthcare emergencies.

## Methods

We designed and conducted an online survey of FLHCWs in nine countries between September–October 2020. We developed the questionnaire in conjunction with a group of expert advisors from Viatris (formerly Upjohn, a Pfizer division), the World Organization of Family Doctors (WONCA), the International Alliance of Patients’ Organizations (IAPO), and the International Pharmaceutical Federation (FIP) with reference to previous surveys conducted during disease outbreaks or natural disasters (Balicer *et al.*, 2006; Chudasama *et al.*, 2020; Kaufman *et al.*, 2012; World Health Organisation, 2020b). Potential end-users, i.e., physicians and pharmacists provided feedback on the face validity of the survey. The survey and the invitation to participate was made available in English, Thai, Arabic, Portuguese, and Spanish. Surveys were translated by health professionals working for Viatris who were either fluent or native speakers in the respective language. Due to scheduling limitations, we deployed the survey in different countries in rolling recruitment across the study period. We obtained ethical approval from the Auckland Health Research Ethics Committee (Ref: AH3064).

### Eligibility criteria

FLHCWs were eligible to participate if they worked as a physician or community pharmacist involved in the healthcare of patients with NCDs and resided in one of the nine selected countries. For the purpose of the questionnaire, we were interested in the following NCDs: diabetes, high cholesterol, cardiovascular disease, depression, and anxiety.

We recruited respondents from the following three regions and nine countries (or collection of states): South East Asia (ASEAN): Malaysia, Thailand, Philippines; Africa and Middle East (AFME): Gulf Cooperation Council, Egypt, South Africa; and Latin America (LATAM): Brazil, Mexico, and Argentina. The countries were pragmatically selected from a group of countries fitting an archetype defined by high NCD burden and varying degrees of readiness to combat NCDs (Ray *et al.*, 2020). Most were LMICs, but we included the Gulf Cooperation Countries, high-income countries, that have a high NCD burden and poor preparedness to combat NCDs, consistent with the LMIC archetype (Ray *et al.*, 2020), recognising that there is diversity within this group of countries in the public health response to COVID-19 (Hale, 2021).

We recruited respondents through Upjohn’s and FIP’s databases of FLHCWs and their offices and partner organizations in each country. Potential respondents were emailed an invitation to participate, together with a link to the survey, which was constructed using REDCap (Copyright © 2020 Vanderbilt University, Nash, TN, USA.).

The survey collected demographic information (age group, sex, country, and state) as well as information about the participant’s role as a FLHCW and the nature of their clinic or practice. Survey questions were framed around three domains: the impact of COVID-19 pandemic on clinical practice and NCDs; barriers to clinical care during the COVID-19 pandemic; and innovative responses to the challenges of the pandemic. The survey consisted of multichoice questions, checkboxes, and Likert-scales and took 5–10 min to complete (see Appendix 1).

### Statistical analysis

We used SAS version 9.4 (Copyright © SAS Institute Inc., Cary, NC, USA.) for data analyses, summarizing demographic information as frequencies for categorical variables, by participant type (physician or pharmacist), and region. Variables of interest were compared using chi-squared tests to assess statistical significance, with *P* = 0.05 used as the cutoff.

## Results

Between September 21 and November 2, 2020, 1347 individuals responded to the survey. Of these respondents, 71.1% were physicians, 531 (39.4%) of whom identified as general practitioners, family physicians, or family medicine specialists, while 420 (31.2%) identified as “Other medical specialist”. Three-hundred and eighty-seven survey respondents (28.7%) identified as pharmacists (Table 1). Most respondents came from the LATAM region (70.5%). The majority of respondents from the GCC were from Kuwait (44.3%) and Saudi Arabia (31.2%).

**Table 1.** Demographic characteristics of physicians and pharmacists summarized by frequency (%)

	Physician (*N* = 951)	Pharmacist (*N* = 387)	Overall (*N* = 1347)
**Age**			
20-39	243 (25.6)	270 (69.8)	515 (38.2)
40-59	438 (46.1)	93 (24.0)	536 (39.8)
60+	269 (28.2)	24 (6.2)	293 (21.8)
**Gender**			
Male	487 (51.2)	149 (38.5)	639 (47.4)
Female	461 (48.5)	236 (61.0)	701 (52.0)
**Region**			
ASEAN	71 (7.5)	50 (12.9)	123 (9.2)
AFME	122 (12.8)	140 (36.2)	264 (19.6)
LATAM	753 (79.2)	195 (50.4)	950 (70.5)
**Type of practice**			
Privately owned practice	428 (45.0)	198 (51.2)	630 (46.8)
Government owned practice	259 (27.2)	18 (4.7)	278 (20.6)
Commercially owned practice	65 (6.8)	34 (8.8)	100 (7.4)
Community-owned or NGO-owned practice	18 (1.9)	57 (14.7)	75 (5.5)
District or regional hospital	71 (8.5)	17 (4.4)	98 (7.3)
Secondary/tertiary hospital	97 (10.2)	17 (4.4)	114 (8.5)
**Practice setting**			
Rural	58 (6.1)	85 (22.0)	145 (10.8)
Urban	887 (93.3)	293 (75.7)	1184 (87.9)
**Currently seeing patients**			
In person	466 (49.0)	248 (64.1)	717 (53.2)
Remotely	65 (6.8)	8 (2.1)	74 (5.5)
Both	387 (40.7)	116 (30.0)	505 (37.5)
Unable to see patients	31 (3.3)	9 (2.3)	40 (3.0)

Almost two-thirds of respondents (61.6%) were over the age of 40, with slightly more female (52.0%) than male respondents (see Table 1). Nearly half of respondents worked in a privately owned practice or clinic and 20.6% in a government-owned establishment. Most respondents (87.9%) worked in urban settings. Pharmacists were younger than physicians and comprised a larger proportion of female respondents.

Most respondents reported that they were seeing patients in-person (53.2%) or seeing patients both in-person and remotely (e.g., via a telemedicine connection) (37.5%). Only a small proportion of FLHCWs reported that they *only* saw patients remotely (5.5%) or were *unable* to see patients due to public health restrictions (3.0%). Fewer physicians reported currently seeing patients in-person (49.0%) compared to pharmacists (64.1%). The proportion of respondents seeing patients in-person was larger in AFME (58.0%) compared to the ASEAN region (49.6%) and LATAM region (52.3%).

### Clinical practice

**Figure 1.**
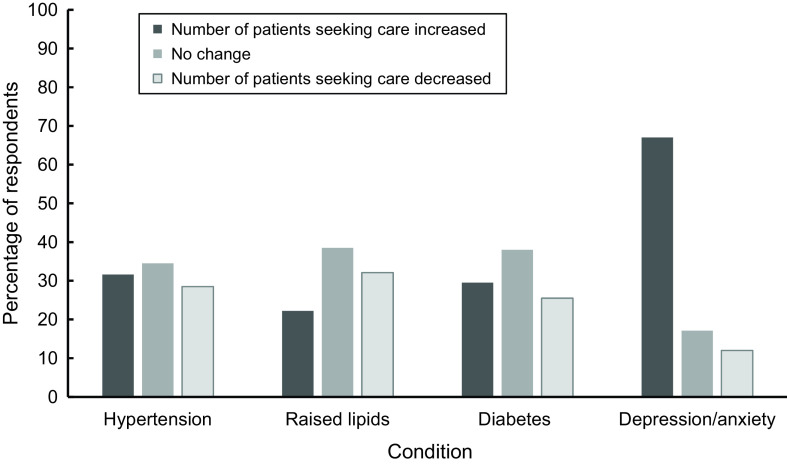
FLHCP perceived change in the number of patients seeking care relating to NCDs or mental health.

Most respondents (93.3%) believed that COVID-19 had adversely affected the mental health of most or some of their patients. The proportion of respondents that answered affirmatively was high across all FLHCWs (>88%), and there was no significant difference between the types of FLHCW. Since the pandemic, more than half of the clinicians perceived an increase in the number of patients seeking care for anxiety and/or depression (67.0%), while findings were mixed for hypertension, raised lipids, and diabetes (Figure 1). Of those who reported an increase in patients seeking care, 23.6% reported that the change was an increase in new patients while 18.6% reported that the change was in existing patients seeking more care; the majority reported that it was a combination of both.

Compared to physicians, a higher proportion of pharmacists reported an increase in patient visits for NCDs, especially hypertension (38.5% of pharmacists versus only 29.0% of physicians). Thirty-seven per cent of physicians reported a decline in the number of patient visits for raised lipids, while only 18.6% of pharmacists reported the same. More physicians reported an increase in patient visits for depression and/or anxiety compared to pharmacists (70.7% of physicians versus 58.7% of pharmacists). Depression and/or anxiety was the only condition for both groups where the majority reported an increase in the numbers of patient visits.

**Figure 2.**
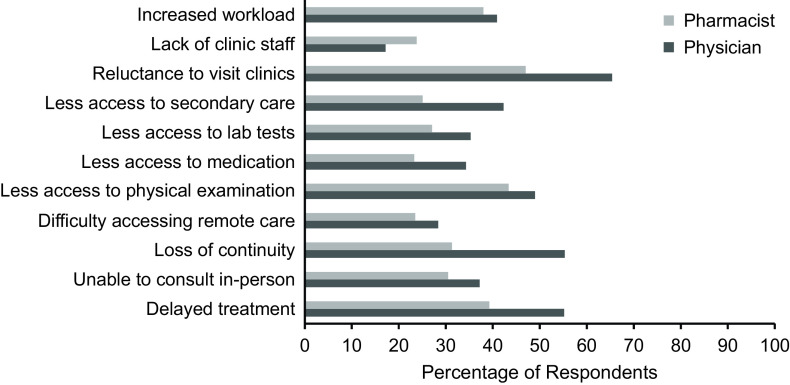
FLHCP perceptions of COVID-19-related factors influencing care of patients.

When asked about the effect that COVID-19 had on the ability to provide healthcare for patients with NCDs, nearly one-third (29.0%) reported that healthcare had improved, while 38.6% reported that care had deteriorated for NCDs. When asked about the effect of COVID-19 on the ability to provide care for patients with depression and anxiety, 36.1% reported that care had improved, while 28.6% reported that care had deteriorated. There were significant differences in the findings between healthcare workers for both NCDs and mental health (*P* < 0.001). As seen in Table 1, a higher proportion of pharmacists than physicians reported improved care for both NCDs and anxiety and depression. Nearly a half of physicians said that their ability to provide care for NCDs had deteriorated, while only a quarter of pharmacists said the same. For care of anxiety or depression, similar proportions of physicians reported that care had deteriorated (33.5%), and that care had improved (31.5%), while 47.8% of pharmacists reported improved care. There was no significant difference in these responses between regions (Figure 2).

For most respondents, clinical or patient care activities had been somewhat or moderately adversely impacted by COVID-19 public health measures (56.2%). A third of respondents reported they had been significantly impacted (35.3%). Only a small proportion (6.8%) reported no impact of public health measures. The highest proportion of respondents reporting no impact was from AFME and lowest from ASEAN (8.7% vs 4.1%, respectively), likely reflecting country-to country variation in the intensity and scale of public health measures (Hale, 2021).

There was a significant difference in the impact of public health measures on pharmacists and physicians (*P* < 0.001). Fewer pharmacists reported that they had been impacted by public health measures and were more than twice as likely to note that public health measures had 'not at all’ impacted their ability to provide healthcare, compared to physicians (11.4% vs 5.0% respectively, Table 2). Only 23.5% of pharmacists reported that they had been significantly impacted compared to 40.1% of physicians.

**Table 2.** Impact of COVID-19 on respondents’ ability to care for patients with NCDs and depression or anxiety

	GP (*N* = 951)	Pharmacists (*N* = 387)	*P* value
**Care of patients with NCDs**			
Care has improved	199 (21.1)	187 (48.6)	
No change in care	309 (32.7)	98 (26.1)	
Care has deteriorated	428 (45.1)	88 (23.7)	
			<0.0001
**Care of patients with depression or anxiety**			
Care has improved	299 (31.5)	184 (47.8)	
No change in care	320 (33.9)	121 (31.3)	
Care has deteriorated	317 (33.5)	63 (16.5)	
			<0.0001
**Extent to which care was impacted by public health measures**			
Not at all	48 (5.1)	44 (11.4)	
Somewhat impacted	219 (23.1)	142 (36.7)	
Moderately impacted	296 (31.3)	96 (24.8)	
Significantly impacted	379 (40.1)	89 (23.5)	
			<0.0001

### Barriers

#### Factors influencing the healthcare of patients

When asked which factors relating to COVID-19 were influencing their care of patients, the most common option was the reluctance of patients to visit healthcare settings during the pandemic (59.8%). The next most common responses were delayed treatment for non-COVID-19 conditions (50.5%), loss of continuity of care (48.4%), and less access to physical examination and monitoring (47.2%). Lack of clinical staff was the least frequently selected option (19.2%).

Although the most frequently endorsed barriers were similar across physicians and pharmacists, the level of endorsement was higher across all factors except for lack of clinical staff, which was more widely endorsed by pharmacists. Most physicians selected delayed treatment (55.2%) and loss of continuity of care (55.3%) as factors influencing healthcare. While delayed treatment was selected by nearly 40% of pharmacists, loss of continuity was only endorsed as a factor influencing healthcare by 31.3% of pharmacists. Pharmacists endorsed lower access to physical monitoring to a lesser extent compared to physicians (43.4% vs 49.0%, respectively), although this was still the second most common factor. Both groups reported the importance of increased workload. Lack of staff was endorsed more frequently by pharmacists than physicians (23.8% vs 17.2%, respectively). Compared to physicians, fewer pharmacists reported less access to medication as a factor influencing care. We found significant differences between the proportions of physicians and pharmacists for the following response options: delayed treatment (*P* < 0.001), inability to have in-person visits (*P* = 0.0128), loss of continuity of care (*P* < 0.001), less access to medication (*P* < 0.001), less access to laboratory tests (*P* = 0.0035), less access to secondary care (*P* < 0.001), a reluctance of patients to visit clinics (*P* < 0.001), and a lack of clinic or pharmacy staff (*P* = 0.007).

#### Barriers to accessing routine care

When asked about the barriers they believed patients were experiencing in accessing routine care during the pandemic, fewer than 1% of respondents considered that there were no barriers. The barrier most frequently endorsed was fear of contracting COVID-19 in healthcare settings (76.7%). The barriers most frequently reported, after fear of contracting COVID-19, were restriction of movement (58.4%) and financial hardship or loss of insurance (55.5%). While fear of contracting COVID-19 in healthcare settings was consistently found across both pharmacists and physicians, this barrier was endorsed by less than two-thirds of pharmacists (61.2%) compared to most physicians (82.5%). Similarly, for financial hardship or loss of insurance, this barrier was strongly endorsed by both groups but to a lesser degree by pharmacists (58.5% of physicians vs 48.6% of pharmacists). Restriction of movement and lack of transport were barriers endorsed more frequently by pharmacists compared to physicians.

### Technology

Most respondents found technology to be useful in managing routine healthcare of NCDs during the pandemic (76.5%). This finding was consistent across regions and type of FLHCW. Most respondents reported that there had been an increase in the number of patients interested in or purchasing devices related to self-care or self-monitoring of NCDs (67.4%). The proportion of respondents that reported an increase was greater for pharmacists (76.7%) than physicians (63.9%). When asked what healthcare practices they would personally be interested in adopting following the COVID-19 crisis, the most frequently endorsed options were patient counselling (58.9%) and preventative activities (58.1%). We observed significant differences across regions in the degree to which the adoption of different technologies was supported. Respondents from the ASEAN region endorsed technologies to a greater extent than the other regions. For example, a significantly higher proportion (90.1%) of respondents from the ASEAN region considered that there would be increased use of teleconsulting or virtual consulting beyond the pandemic, compared to 77.7% and 70.2% in the LATAM and AFME regions, respectively (*P* < 0.001). Respondents from the AFME region generally were less likely to endorse the ongoing use of technology than both the ASEAN and LATAM region.

## Discussion

Our study has revealed that the COVID-19 pandemic has had a range of impacts on the care of patients with NCDs in LMICs by FLHCWs, from constraints on access to care to health effects, in particular, depression and anxiety as noted in other surveys (Ozawa *et al.*, 2019). Despite the challenges, most FLHCWs continue to see patients either in person or remotely, the latter mode is especially common for physicians; pharmacists continue to see patients in-person. Physicians’ ability to conduct physical examinations and monitoring is likely to be a factor in this difference, whereas pharmacists can conduct their work while maintaining social distancing (International Pharmaceutical Federation, 2020). The ability of pharmacists to continue to see patients in-person, in some cases even extending their hours (Aruru *et al.*, 2020), highlights the potential for pharmacists in closing treatment gaps and maintaining contact between patients and the primary healthcare system during crises and emergencies (Basu, 2020, Rasheed *et al.*, 2019). Despite continued access, either remotely or face-to-face, most respondents reported that their ability to provide quality healthcare had been compromised by pandemic control measures.

Adaptation and innovation using available technology was common. FLHCWs strongly supported the greater use of teleconsulting after the COVID-19 pandemic has abated. However, most respondents in our survey were in urban settings and therefore more likely than their counterparts in rural areas to be supported by the robust communications infrastructure needed to support telemedicine.

Our findings also suggest that the ability of FLHCWs to provide care for patients with mental health conditions was less adversely impacted than their ability to provide care for NCDs. One reason that may account for this difference is that the care of patients with depression and anxiety does not require a physical examination (Langarizadeh *et al.*, 2017), and is, therefore, more amenable to telehealth using readily available technology. The increasing availability of remote monitoring technology (such as blood pressure monitors) will likely facilitate physicians in their adoption of telehealth practices in the treatment of NCDs. This is complemented by a movement which is empowering patients to manage their own health and health data (Devi *et al.*, 2020).

The findings of the most common barriers and factors influencing care reported by FLHCWs were important to note. Although a fear of contracting COVID-19 is specific to the current circumstances, the other factors identified (such as restricted movement and financial loss) are consistent with research that has identified barriers to accessing routine care during natural disasters (Arrieta *et al.*, 2008, Balicer *et al.*, 2006, Jaakkimainen *et al.*, 2014).

### Strengths and limitations

To the best of our knowledge, this is the first study that has elicited perceptions from FLHCWs on the impact of COVID-19 on the care of patients with NCDs in LMICs and countries with high NCD burden fitting the LMIC archetype. Uniquely, our survey included pharmacists as FLHCWs. Pharmacists are an important healthcare provider with varied roles across many LMICs, (Miller and Goodman, 2016, Yemeke *et al.*, 2020, Ozawa *et al.*, 2019, Kretchy *et al.*, 2021) and should be included in all such surveys.

Where there were high numbers of participants in particular countries, sufficient statistical power was available to undertake detailed analyses by location and type of FLHCW. However, there were some limitations. First, we used an online survey to collect data, which relied on self-report and perceptions. Second, sampling was nonrandom, convenience, and opportunistic, which limits the generalizability of the findings. We approached existing networks of healthcare providers and may have missed responses from some groups. Third, while we initially aimed to sample 250 respondents from each group (physicians, pharmacists) in each country, we did not meet this target, and we oversampled physicians in Mexico, which may skew findings, particularly given the high number of general practitioners responding in LATAM. The variable number of respondents per country and per occupation meant that individual responses could not be averaged to give country-level responses for cross-country comparisons. Finally, we did not distinguish between the different types of teleconsulting or virtual consulting in the survey questions, limiting our ability to determine which virtual consulting methods (call, video call, text messaging, etc.) were most preferred between the FLHCWs.

### Implications

Greater support is needed for FLHCWs in LMICs who care for growing numbers of patients with depression and anxiety (Torales *et al.*, 2020). Community pharmacists could play a greater role in NCD and mental health management in pandemic situations because patients were less reluctant to visit their premises than attend medical clinics. One proposed solution to improve healthcare accessibility in LMICs is to train community healthcare workers like pharmacists in testing and screening activities that are traditionally performed by physicians, thereby “task shifting” some of the burden from the physicians (Ganju *et al.*, 2020). A reconfiguration of the healthcare system that maximizes pharmacist involvement in NCD care could also help ease the pressure on physicians in providing care during the COVID-19 pandemic in LMICs. Greater attention should be given to increasing technical support for FLHCWS in LMICs to use readily available communications technologies and tools to enable continuity of preventive care and treatment for their patients and communities, particularly in mental health (Dowrick *et al.*, 2020).

## Conclusion

Our findings reiterate the need to ensure continuity of care for NCDs as part of pandemic preparedness so that chronic conditions are not exacerbated by public health measures and the direct impacts of the pandemic.

## Appendix 1. Questionnaire

**Global Survey: Chronic Illness Care and COVID19**

^*^Branching logic in italics

**Table:**

#	Question	Field Format
Section 1**: Demographic Information**		
1.01	Age	• 20–29 • 30–39 • 40–49 • 50–59 • 60–69 • 70+
1.02	Sex	• Male • Female • Other
1.03a	Country	• Malaysia • Thailand • Philippines • Gulf Cooperation Council (Saudi Arabia, UAE, Bahrain, Kuwait, Qatar, Oman) • Egypt • South Africa • Brazil • Mexico • Argentina
1.03b	Region/State *(specific to each country)*	
1.04	What is your role as a frontline healthcare worker?	• General practitioner/family physician/family medicine specialist • Other medical specialist • Pharmacist
1.05	What qualification/specialty do you have? (check all that apply)	• Medical degree • General practice/family medicine • Internal/general medicine • Psychiatry • Cardiology • Endocrinology • Pharmacy degree
1.06	What type of practice/clinic/pharmacy is your *main* place of work?	• Privately owned practice/clinic • Government-owned practice/clinic • Commercially owned practice/clinic (large corporate) • Community-owned practice/clinic • NGO-owned practice/clinic • District hospital • Regional hospital • Secondary/tertiary hospital
1.06a	*If ‘pharmacist’ selected in 1.04* How would you classify your workplace/role?	• Owner operator of community pharmacy • Work for owner of community pharmacy • Work for a chain pharmacy • Pharmacist at a hospital • Clinical pharmacist on a ward • Head of pharmacy at a NGO clinic
1.07	Is your primary practice/clinic located in an urban or rural setting?	• Rural • Urban
1.08	How are you currently seeing patients?	• In person • Remotely (e.g. video consults, telephone consults, email, messaging, home delivery of medicine/devices), • Both remotely and in person • Unable to see patients due to public health restrictions
**Section 2: Clinical Practice**		
	Question	Rationale
2.01	Prior to the COVID-19 pandemic, what proportion of your patients would you say had or were provided treatment for cardiovascular diseases (including hypertension and raised lipids) and/or diabetes?	• Less than 20% • 21%–40% • 41%–60% • 61%–80% • More than 80%
2.02	Prior to the COVID-19 pandemic, what proportion of your patients would you say had or were provided treatment for depression or anxiety?	• Less than 20% • 21%–40% • 41%–60% • 61%–80% • More than 80%
2.03	Since the COVID-19 pandemic, has there been any change in the number of patients that seek care relating to NCDs or mental health?	
2.03a	Hypertension	• Number of patients seeking care increased • No change in number of patients seeking care • Number of patients seeking care decreased
2.03b	Raised lipids	• Number of patients seeking care increased • No change in number of patients seeking care • Number of patients seeking care decreased
2.03c	Diabetes	• Number of patients seeking care increased • No change in number of patients seeking care • Number of patients seeking care decreased
2.03d	Depression and/or anxiety	• Number of patients seeking care increased • No change in number of patients seeking care • Number of patients seeking care decreased
2.03e	*Show if respondent select “increase”* If there has been an increase, has the increase been in new patients or existing patients?	• Increase in new patients • Existing patients seeking more care • Both
2.04	To what extent do you believe that COVID-19 has adversely affected the mental health of your patients?	• Yes – most patients affected • Yes – some patients affected • No – stayed the same, • No – mental health improved
2.05	Has there been any change in the severity of the condition of patients seeking care for NCDs or mental health?	• Severity of the condition of patients has increased • No change in severity • Severity of the condition of patients has decreased
2.06	What effect has the COVID-19 pandemic had on your ability to provide care to your patients with NCDs?	• Care has improved • No change in care, • Care has deteriorated
2.07	What effect has the COVID-19 pandemic had on your ability to provide care to your patients with depression or anxiety?	• Care has improved • No change in care • Care has deteriorated
2.08	To what extent has your clinical or patient care activities been limited by public health measures (e.g. lockdown/quarantine, use of PPE, travel restrictions, etc.) related to COVID-19?	• Not at all • Somewhat impacted • Moderately impacted • Significantly impacted
2.09	Overall, have you observed any changes in your patients’ self-care during the COVID-19 pandemic?	• Increased • No change • Decreased • Don’t know
2.10	Which of the following factors relating to the COVID-19 pandemic are influencing care of your patients? (check as many as apply)	• Delayed treatment for non-COVID-19 conditions • Unable to have in-person consultations with you • Loss of continuity of care • Difficulty accessing at-distance (e.g. phone or video) care • Less access to physical examination and monitoring such as blood pressure and weight • Less access to medications (e.g. shortages) • Less access to laboratory tests • Less access to secondary care referrals • Reluctance of patients to visit healthcare settings during pandemic • Lack of clinic/pharmacy staff • Increased workload due to COVID-19 response measures
2.11	What barriers, if any, do you believe that patients may experience in accessing routine care during the COVID-19 pandemic? (Check all that apply)	• Restriction of movement • Financial hardships or loss of insurance • Lack of transport • Lack of childcare • Clinic closures • Lack of access to healthy food • Medication rationing • Medication shortages • Physical displacement • Lack of communication infrastructure • Unrelated health burdens • Fear of contracting COVID-19 in healthcare settings • No barriers • Other barriers
**\Section 3: Innovation**		
	Question	Field Format
3.01	What technology innovation changes do you think will be likely following the COVID-19 pandemic? (check all that apply)	• Increased use of teleconsulting/virtual consulting • Increased use of electronic prescriptions • E-therapy, digital therapeutics • Use of wearable monitors • Mobile health for self-management • None of the above
3.02	What healthcare practices would you be interested in adopting following the COVID-19 pandemic?	• Patient counselling • Adherence management • Cardiovascular risk assessment • Medication management • Preventative activities • Teleconsulting/Virtual consulting • None of the above
3.03	How useful did you find technology in managing routine care of NCDs during the COVID-19 pandemic?	• Not useful at all • Not useful • Neither useful nor not useful • Useful • Very useful
3.04	Has there been any change in the number of patients interested in or purchasing devices related to self-care or self-monitoring of NCDs such as glucometers, blood pressure monitors, oximeters?	• Increased • No change • Decreased

*Thank you for participating in this survey.*
